# Growth and Morbidity of Gambian Infants are Influenced by Maternal Milk Oligosaccharides and Infant Gut Microbiota

**DOI:** 10.1038/srep40466

**Published:** 2017-01-12

**Authors:** Jasmine C. C. Davis, Zachery T. Lewis, Sridevi Krishnan, Robin M. Bernstein, Sophie E. Moore, Andrew M. Prentice, David A. Mills, Carlito B. Lebrilla, Angela M. Zivkovic

**Affiliations:** 1 Department of Chemistry, University of California, Davis, CA 95616, United States; 2 Foods for Health Institute, University of California, Davis, CA 95616, United States; 3 Department of Food Science and Technology, University of California, Davis, CA 95616, United States; 4 Department of Nutrition, University of California, Davis, CA 95616, United States; 5 Department of Viticulture and Enology, University of California, Davis, CA 95616, United States; 6 Department of Anthropology, University of Colorado, Boulder, CO 80309, United States; 7 Health and Society Program, Institute of Behavioral Science, University of Colorado, Boulder, CO 80309, United States; 8 Medical Research Council (MRC) Human Nutrition Research, Cambridge, UK; 9 MRC Unit, The Gambia and MRC International Nutrition Group, London School of Hygiene & Tropical Medicine, London, UK

## Abstract

Human milk oligosaccharides (HMOs) play an important role in the health of an infant as substrate for beneficial gut bacteria. Little is known about the effects of HMO composition and its changes on the morbidity and growth outcomes of infants living in areas with high infection rates. Mother’s HMO composition and infant gut microbiota from 33 Gambian mother/infant pairs at 4, 16, and 20 weeks postpartum were analyzed for relationships between HMOs, microbiota, and infant morbidity and growth. The data indicate that lacto-*N*-fucopentaose I was associated with decreased infant morbidity, and 3′-sialyllactose was found to be a good indicator of infant weight-for-age. Because HMOs, gut microbiota, and infant health are interrelated, the relationship between infant health and their microbiome were analyzed. While bifidobacteria were the dominant genus in the infant gut overall, *Dialister* and *Prevotella* were negatively correlated with morbidity, and *Bacteroides* was increased in infants with abnormal calprotectin. Mothers nursing in the wet season (July to October) produced significantly less oligosaccharides compared to those nursing in the dry season (November to June). These results suggest that specific types and structures of HMOs are sensitive to environmental conditions, protective of morbidity, predictive of growth, and correlated with specific microbiota.

Breast milk provides nourishment for the developing human infant, but also delivers an assortment of bioactive molecules with important developmental and protective functions. Free human milk oligosaccharides (HMOs) are abundant components of human milk, with over two hundred identified structures so far^1^,^2^. HMOs are composed of glucose (Glc), galactose (Gal), N-acetylglucosamine (GlcNAc), fucose (Fuc), and N-acetylneuraminic acid (Neu5Ac) or sialic acid. HMOs contain a lactose (Gal(ß1-4)Glc) core, and enzymes can transfer monosaccharides onto that core with various linkages^3^. HMOs are not synthesized from a template but are instead formed by the action of competing glycosyltransferases, which transfer monosaccharides to form diverse branched and linear structures, complicating compositional oligosaccharide analysis^3^. For example, the structure lacto-*N*-tetraose (LNT, Gal(ß1-3)GlcNAc(ß1-3)Gal(ß1-4)Glc) is fucosylated to form lacto-*N*-fucopentaose I (LNFP I, Fuc(α1-2)Gal(ß1-3)GlcNAc(ß1-3)Gal(ß1-4)Glc). HMOs provide a number of benefits to infants, including brain development^4^, acting as decoys for pathogenic bacteria^5^, and preventing disease and infection^6^.

HMOs are not digestible by infants and arrive intact to the large intestine, where they play an important role in the development of the gut microbiota by nourishing specific bacteria (i.e. acting as prebiotics)^7^. Select *Bifidobacterium* and *Bacteroides* species have been shown to consume HMOs^8^. Infants with bifidobacteria-dominated gastrointestinal tracts have higher resistance to colonization by some pathogens and enhanced gut barrier function^9^,^10^,^11^,^12^. Bifidobacteria aid the development of the infant’s innate and acquired immune systems, enhancing surveillance and reducing inflammation^12^,^13^,^14^,^15^. Some bifidobacteria are better equipped for HMO consumption than others. *Bifidobacterium longum* subsp. *infantis* was found to better colonize premature infant guts than *Bifidobacterium animalis* subsp. *lactis* when given concurrently with human milk, likely due to the capacity of *B. longum* subsp. *infantis* to consume a wide spectrum of HMOs^16^,^17^,^18^,^19^,^20^. *B. longum* subsp. *infantis* colonization is associated with improved responses to some vaccines, decreased intestinal epithelial permeability, and has anti-inflammatory effects on the premature intestine^10^,^12^,^21^.

The effects of HMOs on health are composition- and structure-specific, and HMO composition varies between mothers and over time^6^,^22^,^23^,^24^. One quality that varies between mothers and has been shown to influence the microbiota and health of infants is secretor status^25^,^26^. Secretors are individuals who secrete ABH antigens in their bodily fluids and can link Fuc in an α(1–2) position on a terminal Gal residue. The α(1-2) fucosyltransferase is responsible for this linkage and is encoded for by the secretor locus^27^,^28^; this genotype can be observed phenotypically by analyzing the HMO composition of milk^27^. Notably, secretor mothers have higher relative concentrations of fucosylated HMOs, specifically those with α(1-2) Fuc residues including 2′-fucosyllactose (2′FL), lacto-*N*-fucopentaose I (LNFP I), lactodifucotetraose (LDFT), and difucosyllacto-*N*-hexaose a (DFLNHa), but lower relative concentrations of undecorated and sialylated HMO^27^.

The Gambia provides a special opportunity to observe how large fluctuations in environmental factors due to seasonality affect oligosaccharide composition in breast milk and the microbial profile in the infant gastrointestinal tract. Due to strong seasonal differences in climate, Gambians experience two distinct seasons during the year, a dry season and a wet season. During the dry (“harvest,” November to June) season, food is plentiful, energy stores are higher, and disease burden is relatively low; however, during the wet (“hungry,” July to October) season, food stores become depleted, rates of infection increase, and farming workload increases. The effects of this seasonal dichotomy on health and physiology have been documented across several decades of work in rural Gambian populations^29^. In particular, patterns of weight loss and breast milk output link very closely to the seasonal shifts in workload, food availability, and disease burden^30^. Similarly, Gambian infants show clear seasonal patterns of morbidity, like skin infections and diarrhea, which are much higher during the wet season compared to the dry season^31^,^32^. Higher rates of morbidity can affect infant growth by redirecting bodily resources toward fighting infection or repairing damage rather than growth. By profiling breast milk at three early time points during lactation when breast milk is the predominant or sole source of nutrition to the infant, it is possible to assess the relationship between seasonal changes in HMO composition and the infant’s microbiome, growth, and morbidity status (whether the infant was “sick” or “not sick”, as defined in the Materials and Methods section).

In this study, the HMO composition in the breast milk and the infant gut microbiota of 33 rural Gambian mother/infant pairs were profiled at 4, 16, and 20 weeks postpartum and related to growth and morbidity in the infants. We also examined HMO composition and gut microbiota to investigate if shifts in milk glycan composition correlate with the gut microbial community. The aim of this work was to determine whether HMO content and composition are influenced by seasonal environmental changes, and whether changes in HMO composition are associated with protection from morbidity, improved growth, and with the intestinal microbiome.

## Results

### Changes in HMOs and microbiota over time

**Figure 1:**
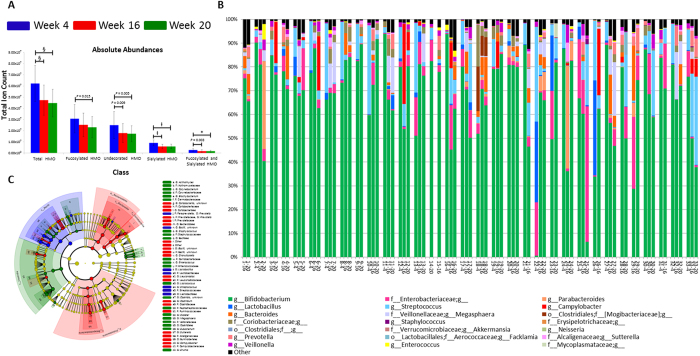
Changes over time in human milk oligosaccharides (HMOs) and microbiota. (**A**) Changes in human milk oligosaccharides (HMOs) and microbiota from week 4 (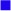) to week 16 (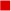) to week 20 (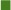) postpartum. Absolute abundances of HMOs show a decrease for all classes: total oligosaccharides, total fucosylated HMO, undecorated HMO, total sialylated HMO, and fucosylated and sialylated HMO. *P*-values for differences between time points are denoted by * for *P* < 0.001, § for *P* < 0.0001, and for *P* < 0.00001 based on linear mixed effects models. Undecorated HMO abundances were log transformed and *P*-values are based on transformed data. (**B**) Microbiota composition for each infant at one or more time points. The most abundant genus across all samples was *Bifidobacterium* (green bar). (**C**) Cladogram of microbiota enriched at each time point with results based on LefSe analysis. Bacilli were enriched at week 4 (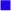), Bacteroidetes, Proteobacteria, and Coriobacteriia were enriched at week 16 (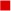), and Clostridia were enriched at week 20 (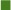).

In order to identify shifts in HMO composition over time, we first measured the oligosaccharide content of mother’s milk at all three time points. The total oligosaccharide amounts significantly decreased from 4 to 20 weeks postpartum (Fig. 1A). Absolute abundances for total HMOs, total fucosylation, undecorated HMOs, total sialylation, and fucosylated and sialylated HMOs were significantly different between weeks 4 and 16 and weeks 4 and 20 (Fig. 1A). Total fucosylation was only statistically different between weeks 4 and 20 (*P* = 0.02) (Fig. 1A). The relative abundances of each class were not statistically different throughout lactation.

**Figure 2:**
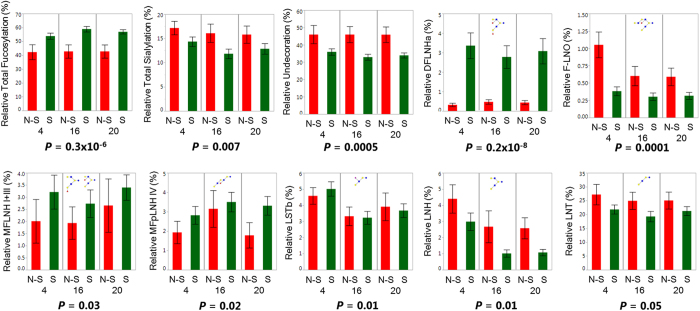
Differences in relative human milk oligosaccharide (HMO) classes and individual structure abundances between secretors and non-secretors. Differences in relative human milk oligosaccharide (HMO) class and structure abundance between phenotypic secretors (S, 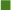, *N* = 21) and non-secretors (N-S, 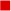, *N* = 12). Plots show averages for all mothers divided by time point (week 4, 16, and 20) with error bars representing standard error and *P*-values listed below each plot for overall differences based on Mann-Whitney U tests. Individual HMO structures pictured with monosaccharides Glc (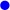), Gal (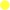), GlcNAc (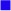), Fuc (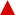) and Neu5Ac (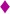).

The individual HMO structures that were quantified in this study are shown in Table 1. Included are their relative abundances at each time point, in how many of the 33 milk samples each structure was found, and changes in abundances over the three time points. For the analyses in this study, relative HMO class abundances and the relative abundances of individual HMOs that were found in at least 75% of all samples during at least two of the three weeks were used. These structures included lacto-*N*-tetraose, LNT; lacto-*N*-fucopentaose I and lacto-*N*-fucopentaose III, LNFP I and LNFP III; lacto-*N*-neotetraose, LNnT; sialyllacto-*N*-neotetraose, LSTc; monofucosyllacto-*N*-hexaose I and monofucosyllacto-*N*-hexaose III, MFLNH I and MFLNH III; fucosyl-para-lacto-*N*-hexaose, MFpLNH IV; sialyllacto-*N*-tetraose (b), LSTb; lacto-*N*-neohexaose, LNnH; difucosyllacto-*N*-hexaose (a), DFLNHa; lacto-*N*-hexaose, LNH; difucosyl-para-lacto-*N*-hexaose II, DFpLNH II; difucosyllacto-*N*-hexaose (b), DFLNHb; 3′-sialyllactose, 3′SL; monosaccharide composition 4 hexose:0 fuc:2 GlcNAc:1 Neu5Ac and monosialyllacto-*N*-neohexaose II, 4021a and S-LNnH II; isomer III fucosyl-paralacto-*N*-hexaose, IFLNH III; and fucosyllacto-*N*-octaose, F-LNO. Several of these structures with known α(1-2) Fuc linkages were used to determine maternal phenotypic secretor status (see Materials and Methods section). In this cohort, 12 of the 33 mothers were determined to be non-secretors, with relative α(1-2) fucosylation less than 6% of total HMOs for non-secretors. The significant differences found in relative class and individual HMO abundances between secretors and non-secretors are shown in Fig. 2. As has been observed before, secretor mothers had higher relative concentrations of total fucosylated HMOs, but lower relative concentrations of undecorated and sialylated HMOs.

Overall, the gastrointestinal tracts of Gambian infants were consistently dominated by bifidobacteria, which represented 68.3% of total bacteria on average (Table 2). Of the 85 samples for which sufficient fecal material was available for microbiome investigation, only two samples contained less than 10% bifidobacteria (2.35% of samples) and only four other samples contained less than 40% bifidobacteria (Fig. 1B). A taxonomic group other than bifidobacteria was the majority bacterial group in only five samples (5.88% of samples) (Fig. 1B). Using *Bifidobacterium-*specific methods (including the BLIR method described in the methods section), we determined that 76.9% of the bifidobacteria in the samples were one subspecies, *B. longum* subsp. *infantis (B. infantis*), making it the most abundant taxon in these Gambian infants (Table 2). The next most abundant bifidobacteria*, B. longum* subsp. *longum,* accounted for 14.1% of the bifidobacteria in these infants (Table 2). Other significant operational taxonomic units (OTUs) in the infants included *Streptococcus* (7.7%), the Enterobacteriaceae family (5.4%), *Megasphaera* (2.4%)*, Bacteroides* (2.0%)*, Campylobacter* (1.8%)*, Lactobacillus* (1.8%), the Coriobacteriaceae family (1.6%), *Parabacteroides* (1.5%)*, Prevotella* (1.2%), and *Veillonella* (1.0%). Individual infant microbial profiles changed over time (Fig. 1B). Even in the presence of a dominant bifidobacterial population, significant changes in community structure were noted in the first weeks of life. For example, the Bacilli class was more abundant at 4 weeks than at other time points, the abundances of the Bacteroidetes phylum, Proteobacteria phylum, and Coriobacteriia class were higher at 16 weeks, and the Clostridia class was enriched at 20 weeks compared to other time points (Fig. 1C).

### Associations with infant morbidity

**Figure 3:**
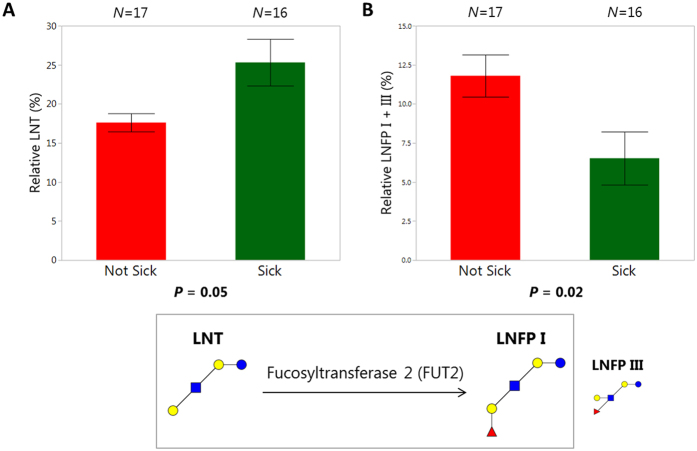
Differences in individual human milk oligosaccharide (HMO) structures between infants who were “sick” or “not sick” at week 16. Differences in individual human milk oligosaccharides (HMOs) between infants who had reported sick days (Sick, 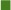) and those who did not (Not Sick, 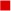). Plots are divided by sick and not sick infants, displaying week 16 average and standard error bars for relative abundances of (**a**) LNT (lacto-*N*-tetraose) and (**b**) LNFP I and III (lacto-*N*-fucopentaose I and lacto-*N*-fucopentaose III). HMO structures pictured with monosaccharides Glc (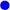), Gal (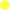), GlcNAc (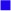), and Fuc (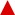) with the enzyme listed for the creation of LNFP I from LNT. *N* for each group are given above the respective groups and *P*-values are listed below each plot based on Mann-Whitney U tests.

In order to compare mothers’ HMO profiles between infants who had sick days with those who did not have sick days (see Supplementary Table 1), Mann-Whitney U with Steel-Dwass *post hoc* tests were performed. Relative abundances for all HMO classes and individual HMOs that were found in at least 75% of all samples during at least 2 of the 3 weeks were compared. Mothers of infants who were sick at week 16 produced milk with higher relative amounts of LNT than mothers with infants who were not sick (*P* = 0.05), but those same mothers with sick infants produced significantly lower levels of LNFP I and III (*P* = 0.02) (Fig. 3). Relative abundances of F-LNO at week 4, MFpLNH IV at week 16, and IFLNH III at week 16 were found to be higher in infants who were sick compared to those who were not sick (see Supplementary Fig. S1). There were no statistically significant differences in HMO profiles in relation to reported morbidity at week 20.

**Figure 4:**
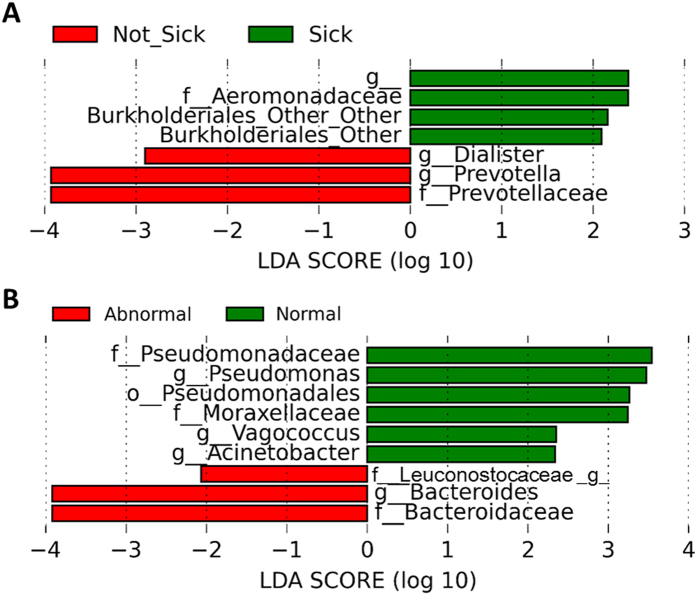
Differences in microbiota between infants who were reported to be “sick” or “not sick” and different calprotectin levels. Plot showing the linear discriminant analysis (LDA) scores for the enriched microbiota based on infant morbidity outcomes. (**a**) Score for microbiota that were found to be enriched in infants who were reported to have sick days (Sick, 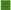), and those who were reported to have no sick days (Not Sick, 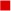). (**b**) Score for the microbiota that were found to be enriched in infants who had normal (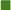) or abnormal (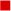) calprotectin levels. Calprotectin categories were created using published cut-offs for normal and abnormal levels.

Similarly, linear discriminant analysis (LDA) effect size (LefSe) analysis was used to determine which bacteria were associated with reported morbidity. Levels of the most abundant taxon, bifidobacteria, were not significantly different between infants who were sick and not sick. Though they were minor members of the community, higher levels of *Dialister* and *Prevotella* were found in infants whose mothers reported their infant was not sick, while increased (but still low) amounts of unidentified Burkholderiales and Aeromonadaceae were associated with sickness (Fig. 4A and Supplementary Fig. S2).

Associations between fecal calprotectin and HMO and the gut microbiota were also assessed. For these analyses, infant calprotectin levels were used as continuous values and also binned into normal (<50 mg/kg), borderline (50–120 mg/kg), and abnormal (>120 mg/kg) categories using published cutoffs^33^,^34^,^35^. Multiple linear regression stepwise models were created with relative class and individual HMO and microbiota to predict infant fecal calprotectin levels at each week. Microbiota with an average relative abundance of at least 1% were also used (*Bifidobacterium, Streptococcus*, the Enterobacteriaceae family, *Megashpaera, Bacteroides, Campylobacter, Lactobacillus*, the Coriobacteriaceae family, *Parabacteroides, Prevotella*, and *Veillonella*). No significant models were found for either HMO or microbiota to predict calprotectin values, whether used as a continuous or categorical value. Calprotectin levels were, however, significantly higher at week 4 than week 16 (see Supplementary Fig. S3).

As the composition of the microbiota would be predicted to influence fecal calprotectin levels, individual bacterial species were screened against the calprotectin class of the infants. Though the abundance of the major bacterial group in these infants (bifidobacteria) did not differ by the infant’s calprotectin level, a few lower abundance species were associated with the calprotectin level. Several members of the Pseudomonadales order (*Acinetobacter* and *Pseudomonas*) and *Vagococcus* were associated with normal (low) fecal calprotectin levels, while unidentified Leuconostocaceae and *Bacteroides* were associated with abnormal (high) fecal calprotectin levels (Fig. 4B and Supplementary Fig. S4).

### Associations with infant growth

Weight-for-age Z scores (WAZ) and height-for-age Z scores (HAZ) were calculated against a Gambian reference for the infants at all time points (see Supplementary Table 2). AUC over the 4, 16, and 20 week time points was calculated using the Trapezoidal rule for relative abundances of HMO classes and the individual structures that were found in at least 75% of samples for at least 2 time points. The ability of the HMO AUCs to predict WAZ and HAZ scores at 20 weeks was determined using multiple linear regression stepwise models. The model for WAZ at 20 weeks showed a significant overall *P*-value of 0.8 × 10^−6^; the AUC for the relative abundance of 3′SL had a positive contribution while LSTc had a negative contribution (Table 3). DFLNHa and LNFP I and III had positive contributions to HAZ at 20 weeks (*P* = 0.05) (Table 3). As shown in the cladogram (see Supplementary Fig. S5), which indicates the microbes enriched in each group colored according to the legend, LEfSe results for the microbiota and infant growth indicate that several members of class Bacilli were enriched in stunted infants, and *Adlercreutzia* and *Klebsiella* were enriched in wasted infants (see Materials and Methods section for binning).

### Associations with seasonal fluctuations

**Figure 5:**
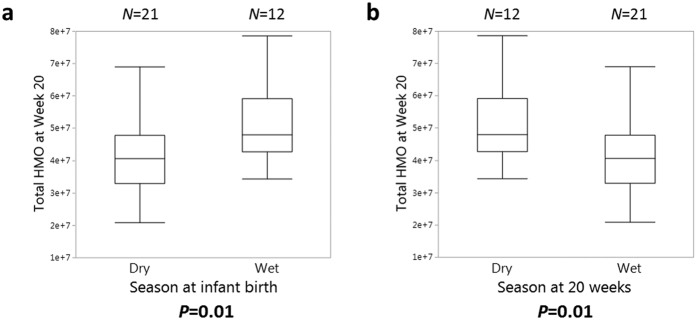
Comparison of mother’s total human milk oligosaccharide (HMO) production by infant birth season and 20 weeks postpartum. Box plots of total human milk oligosaccharides (HMO) production at 20 weeks postpartum compared by (**a**) infant birth season and (**b**) what season mothers were living in 20 weeks postpartum. All mothers who gave birth during the wet season were living in the dry season 20 weeks postpartum, and all mothers who gave birth during the dry season were living in the wet season 20 weeks postpartum. Mothers lactating in the wet season produced less total HMO during that time point compared to mothers lactating in the dry season during that time point. In both plots the median is given as a line, the 25th and 75th percentiles are the top and bottom of the boxes, and the whiskers are data within 1.5*(75th–25th percentile) of the median. *N* for each group are given above the respective groups and *P*-values are listed below each plot based on Mann-Whitney U tests.

Mothers displayed significant variation in their total oligosaccharide abundance based on the season they were nursing in. Mothers who gave birth during the wet season appeared to maintain their total HMO production after 20 weeks of lactation, producing higher amounts of total HMOs than mothers who gave birth during the dry season (Fig. 5A). After 20 weeks postpartum, however, all mothers who gave birth during the wet season were nursing in the dry season and vice versa for those who gave birth during the dry season. Mothers nursing in the dry season produced significantly higher abundances of total HMOs compared to mothers nursing in the wet season (Fig. 5B). Based on LefSe analysis the lower-abundance taxa, Bacilli, *Flexispira*, and *Mollicutes* were more prevalent in babies born in the wet season (see Supplementary Fig. S6).

### Associations between HMOs and infant microbiota

**Figure 6:**
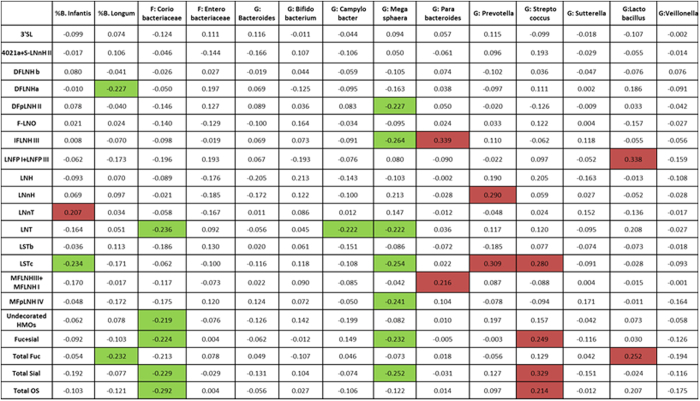
Correlation matrix results of associations among absolute HMO abundances and most prevalent microbiota. Pearson’s correlations between absolute abundances of total human milk oligosaccharides (HMOs), HMO classes (Total Fuc, Total Sial, Undecorated HMOs, and Fuc + sial), and the individual HMOs found in at least 75% of samples for at least two time points (LNT, lacto-*N*-tetraose; LNFP I + LNFP III, lacto-*N*-fucopentaose I + lacto-*N*-fucopentaose III; LNnT, lacto-*N*-neotetraose; LSTc, sialyllacto-*N*-neotetraose; MFLNH I + MFLNH III, monofucosyllacto-*N*-hexaose I + monofucosyllacto-*N*-hexaose III; MFpLNH IV, fucosyl-para-lacto-*N*-hexaose; LSTb, sialyllacto-*N*-tetraose (b); LNnH, lacto-*N*-neohexaose; DFLNHa, difucosyllacto-*N*-hexaose (a); LNH, lacto-*N*-hexaose; DFpLNH II, difucosyl-para-lacto-*N*-hexaose II; DFLNHb, difucosyllacto-*N*-hexaose (b); 3′SL, 3′-sialyllactose; 4021a + S-LNnH II, monosaccharide composition 4 hexose:0 fuc:2 GlcNAc:1 Neu5Ac + monosialyllacto-*N*-neohexaose II; IFLNH III, fucosyl-paralacto-*N*-hexaose III; and F-LNO, fucosyllacto-*N*-octaose), and the most abundant infant microbiota. Infant microbiota data in the first two columns (% *B. infantis* and % *B. longum*) are calculated from a combination of the Bifidobacterial-specific -terminal restriction fragment length polymorphism, *Bifidobacterium* Longum-Infantis Ratio (BLIR), and 16S sequencing analysis as described in the methods section, while microbiota data in the remaining columns are from the 16S sequencing analysis alone. Values highlighted in red (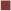) indicate significant positive correlations (*P* ≤ 0.05) and plots highlighted in green (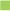) indicate significant negative correlations (*P* ≤ 0.05).

Correlation plots were used to determine the relationships among the HMO abundances and the top 12 most abundant microbiota (along with the relative abundances of *B. infantis* and *B. longum* subsp. *longum* to the fecal microbiota as a whole) (Fig. 6). Irrespective of time, *Prevotella* and *B. infantis* had negative correlations with sialylated structure LSTc. *B. infantis* was the only microbe that was positively correlated with LNnT abundance. *B. longum* subsp. *longum* was negatively correlated with total fucosylation, but *Lactobacillus* had a positive relationship with total fucosylation. *Lactobacillus* also had a positive association with absolute LNFP I and III. *Coriobacteriaceae* and *Megasphaera* had negative correlations with total sialylation, fucosylated and sialylated, and LNT, and *Coriobacteriaceae* also had a negative correlation with total HMOs. *Streptococcus* had positive correlations with total sialylation, fucosylated and sialylated, and LSTc. *Campylobacter* had a negative correlation with LNT. *Parabacteroides* had a positive relationship with MFLNH I and III and IFLNH III.

### Predicting microbiota functions

In order to investigate the potential mechanism by which the above differences in taxonomic profiles might influence community metabolic potential and/or host biology, PICRUSt was used to predict the functional profile of the community. A total of 6909 functions were predicted to be possessed by the combined OTUs in this sample set. After false discovery rate (FDR) correction no functions were found to be significantly different (*P* ≤ 0.05) between infants with different clinical levels of calprotectin, fed by mothers of different secretor status, or reported as sick versus non-sick (data not shown). However, three functions were found to change over early life: K00844: hexokinase [EC:2.7.1.1], K01036: butyrate-acetoacetate CoA-transferase [EC:2.8.3.9], and K01615: glutaconyl-CoA decarboxylase [EC:4.1.1.70] (see Supplementary Fig. S7). On average, the levels of these three functions increased as a proportion of the predicted infant fecal metagenomes over the first 20 weeks of life. The major microbes that were found to contribute these functions (mostly Bacteroidales and Clostridiales such as *Megasphaera*) are shown in Supplementary Table 1.

## Discussion

The results from this study suggest that HMOs consumed by infants and the associated microbiota both impact infant health outcomes. These associations with proxy health measures in the present study comprise context-specific correlations, which at present have only theoretical causational support. However, this study is valuable as an initial hypothesis-generating screen from which to direct future research efforts into mechanisms. The data indicate that mothers of infants who had zero sick days (reported instance(s) of diarrhea, fever, rash, coughing, etc.) produced milk with lower relative amounts of LNT and higher relative amounts of LNFP I and III compared to mothers of infants who had sick days. Isomers LNFP I and III could not be chromatographically separated during analysis, but since LNFP III is <5% the abundance of LNFP I, discussion was focused on LNFP I^5^. LNFP I is the fucosylated version of LNT, with Fuc in an α(1-2) linkage on the terminal Gal. F-LNO at week 4 and MFpLNH IV and IFLNH III at week 16 were also different between sick and not sick infants, but these fucosylated structures were found at higher relative abundance in the sick infants. The Fuc linkage on all three of these structures is α(1-3), unlike LNFP I which is an α(1-2) linkage, and they are found at much lower abundance (less than 5%) than LNFP I which makes up around 12% of HMOs. Total relative fucosylation was not significantly different between sick and not sick infants. Previous studies have also shown that higher consumption of α(1-2) linked Fuc led to lower experiences of diarrhea^26^. Newburg *et al*. showed a decreased incidence of diarrhea in infants who consumed milk with higher ratios of α(1-2) linked Fuc compared to HMOs with α(1-3/4) linked Fuc^26^. Previous studies have found 2′-fucosyllactose (2′FL, Fuc(α1-2)Gal(ß1-4)Glc) to be associated with reduced diarrhea incidence caused by *Campylobacter jejuni*, an infant gastrointestinal pathogen^36^,^37^,^38^. In this study we did not observe any statistically significant associations between reported morbidity and 2′FL. Instead, our results showed that it was the α(1-2) fucosylation of LNT to LNFP I that was associated with lower morbidity. Together, these findings point to specific roles for different α(1-2) fucosylated structures in the protection from morbidity.

Statistical models were used to predict WAZ and HAZ scores at 20 weeks as a response to the HMO abundances. Relative 3′SL (Neu5Ac(α2-3)Gal(ß1-4)Glc) was the strongest model predictor, contributing positively to WAZ, meaning the higher the proportion of 3′SL produced by the mother from 4 to 20 weeks, the larger her infant’s WAZ score at 20 weeks was. LSTc (Neu5Ac(α2-6)Gal(ß1-4)GlcNAc(ß1-3)Gal(ß1-4)Glc), on the other hand, contributed negatively to WAZ at 20 weeks. Relative total sialylation was not a predictor. As with the positive associations between α(1-2) but not α(1-3) fucosylated structures and morbidity, again here, an α(2-3) but not an α(2-6) sialylated species was associated with better growth. Infants from a Malawian cohort who were severely stunted at 6 months postpartum had mothers who produced significantly less sialylated HMO than mothers with infants of healthy height^39^, agreeing with our findings here of a positive association between sialylated HMO and growth. Increased weight gain was also observed in model mice and piglets fed diets with supplemented sialylated bovine milk oligosaccharides, mainly 3′SL and 6′SL (Neu5Ac(α2-6)Gal(ß1-4)Glc)^39^.

In addition to 3′SL, LNFP I was also found to contribute positively to growth, in this case HAZ, at 20 weeks. As indicated earlier, LNFP I was found at higher abundance in the milk of infants who did not have sick days. These findings suggest that LNFP I may help infants maintain growth by sparing resources that would otherwise be redirected toward fighting off infection. Whether these changes in relative concentration of LNFP I are indicative of upregulation of the FUT2 gene, its activity, and/or other compositional changes in milk that are then responsible for protecting the infant from infection, or whether LNFP I directly protects the infant by binding and inactivating pathogens in the intestine, needs to be determined in future studies.

The mothers’ HMO profiles were significantly affected by the seasonal changes experienced in The Gambia. Prentice *et al*. showed that during the dry season, lactating mothers had an increased energy intake compared to mothers lactating in the wet season, which could explain the ability of mothers to produce milk with higher concentrations of HMOs when nursing in the dry season^40^. Previous research has also shown seasonal variation in overall milk output, not just HMO output, with a reduced amount of milk consumption during the wet season, concomitant with a higher risk for infection and growth failure in infants born during the wet season^41^,^42^. HMO production decreases throughout lactation, but there could be a potentially larger decrease in HMO output by mothers nursing in the wet season at 20 weeks postpartum due to their reduced caloric intake. This could be detrimental to their infant’s growth and morbidity status. It is important to note, however, that as the infant grows older he/she consumes a higher total volume of milk per day and therefore the total dose of oligosaccharides per day may remain consistent, although they are consuming a less concentrated milk. Total volume of milk consumed by each infant was not measured in this study. Although the majority of infants were exclusively breast-fed through 20 weeks, the introduction of semisolid foods and water could also affect milk intake and milk composition. The mechanisms by which a seasonal energy deficit in the mother is translated to HMO production levels in the mammary gland are currently unknown and deserve further study to better understand how infant needs are communicated to the mother to alter milk composition to meet those needs.

In this study we chose to analyze the HMO data as relative rather than absolute abundances of HMO, because as noted above, the total amount of milk consumed by each infant each day was not measured, therefore the total abundance of HMO consumed by each infant could not be calculated. However, we also focused on relative abundances because we were interested in how changes in the composition of HMOs affect health outcomes. The compositional differences are indicative of fluxes through the glycosyltransferase pathways. For example, an increased relative concentration of the fucosylated structure LNFP I with a concomitant decrease in the relative concentration of its precursor LNT is indicative of an increased flux in α(1-2) fucosylation. The increased α(1-2) fucosylation of specific HMO in this cohort was associated both with protection from morbidity and with improved growth. Mechanistic studies are needed to understand how seasonal, dietary, environmental, genetic, and/or other factors influence the ability of the mammary gland to turn on α(1-2) fucosylation in an individual mother to increase protection of the infant from infection. Similar mechanisms may be involved in the regulation of the production of sialylated structures and growth.

As observed in other cohorts of infants from less-developed countries like Venezuela, Bangladesh, and Malawi, bifidobacteria (and specifically *B. infantis*) were the dominant microbe in this group of infants^10^,^43^,^44^. *B. infantis* possesses a large genetic complement of enzymatic tools to consume the major human milk glycans in breast milk, suggesting that the dominance of the gut community by this microbe is driven by the availability of these substrates^17^,^19^,^20^. Indeed, in this study, we found that *B. infantis* was the only (sub)species positively correlated with LNnT, which represented ~10.1% of total HMO. The observed shifts in microbial community composition over time (class Bacilli being more abundant at 4 weeks, the Bacteroidetes phylum, Proteobacteria phylum, and Coriobacteriia class more common at 16 weeks, and the Clostridia class more abundant at 20 weeks) may represent the succession of species more acclimated to environmental conditions promoted by weaning from an exclusively breast milk diet, and/or changes in the microbial exposure of the infant as he/she starts to move and sample their environment. Of the 23 infants whose microbiota was measured at all three time points, seven had received water, cow’s milk, and/or semisolid food by week 20. Three infants were also fed from a bottle for some of their meals rather than feeding at the breast, which could also contribute to bacterial diversity at 20 weeks. Initially colonizing Lactobacilli (of possible vaginal origin) present at 4 weeks may be replaced by both *Bacteroides* species able to consume select HMOs and by species that can use the metabolic end products of the dominant bifidobacteria^45^,^46^. A shift toward higher amounts of Clostridia is also associated with the consumption of weaning foods^47^,^48^.

Colonization by bifidobacteria has been associated with proxies for desirable infant health outcomes such as higher resistance to pathogen colonization, improved response to some vaccines, better gut barrier function, enhanced immune surveillance, and reduced inflammation^9^,^10^,^12^,^13^,^14^,^15^. However, we found no correlations between bifidobacteria levels and morbidity, likely because of the ubiquity of bifidobacteria in this cohort. We did find, however, that infants who did not have sick days had higher *Prevotella*, which agrees with a previous study in a cohort of children from Malawi^44^. Although there were too few such cases in this cohort of 33 infants to allow for statistical analysis, several infants had large shifts in the total amount of bifidobacteria at certain time points, which coincided with changes in growth patterns and/or morbidity measures. For example, infant 25 started out with over 60% bifidobacteria at weeks 4 and 16, which fell to less than 10% bifidobacteria and was largely replaced by *Enterobacteriaceae* at week 20. Although she had not had any sick days at either week 4 or week 16, at week 20 this infant had 12 sick days, a very high calprotectin level (867.67 mg/kg), and lost weight from week 16 to week 20. Following a similar pattern, the relative abundance of LNFP I in her mother’s milk went from 24% at week 4 to 18% at week 16, to a very low level of 0.1% at week 20. At the same time, the relative abundance of 2′FL stayed about the same (12%, 12%, 11%), whereas the relative abundance of LNT increased from 15% at week 4, to 26% at week 16 and 23% at week 20. The overall composition of milk decreased 12% in fucosylated structures but increased 10% in undecorated HMOs. Percent sialylation varied from 12% at week 4 to 9% at week 16 to 17% at week 20. In this particular case, the infant was born in the dry season, but mother and infant transitioned into the wet season in week 16 and were still in the wet season at week 20. These findings indicate that significant shifts in the mother’s HMO milk composition coincided with both the shifts in the infant’s gut microbiota and with the infant’s growth and morbidity outcomes (see Supplementary Fig. S8). They also highlight the seasonally-driven shifts in resource availability for mothers, and again point to LNFP I as a potentially important indicator of health outcomes.

Our finding that *Bacteroides* was increased in those infants who had high fecal calprotectin levels is intriguing in light of several recent reports. High levels of *Bacteroides* colonization are common in many of the cohorts of infants from developed countries studied so far, and are more common in vaginally-delivered infants^25^,^49^,^50^. Whereas *B. infantis* is specifically specialized to consume a broad array of HMOs^17^,^51^,^52^, *Bacteroides* species are also able to consume at least some HMOs, though select bifidobacteria outcompete *Bacteroides* for these carbon sources^46^. Our results demonstrate that in the background of a dominant bifidobacterial population, increasing amounts of *Bacteroides* species are associated with increased neutrophil-related gut inflammation as measured by fecal calprotectin levels. Vatanen *et al*. showed that *Bacteroides* presence may expose infants to increased amounts of lipopolysaccharide (LPS) types which are linked to downstream autoimmune disease by inhibiting the immune-stimulating effects of *E. Coli* derived LPS^53^. It is unknown whether the *Bacteroides* species present in the Gambian infants studied here possessed LPS types similar to the *Bacteroides dorei* studied by Vatanen *et al*. *Bacteroides* degradation products of sialylated milk oligosaccharides have been shown to promote the growth of inflammation-inducing (and potentially pathogenic) Enterobacteriaceae in *in vitro* studies, however we did not detect an enrichment of enterobacteria in infants with abnormally high calprotectin levels^39^,^54^,^55^.

Our data show a positive correlation between *Lactobacillus* and fucosylated structures; an observation supported by a study showing that *Lactobacillus casei* BL23 contains genes encoding fucosidases which were capable of degrading fucosylated oligosaccharides^56^. Other lactobacilli, however, such as *Lactobacillus delbrueckii* and *L. rhamnosus* show very little growth on 2′FL^57^. Our findings of positive correlations between *Streptococcus* and total sialylation, fucosylated and sialylated structures, and LSTc match a report that several strains of *Streptococcus* have been shown to produce sialidases^58^. Other streptococci strains, however, are negative for sialidase activity^59^. *B. longum* subsp. *longum* was negatively correlated with fucosylated HMO, confirming previous observations^25^. *B. longum* subsp. *longum* is likely less efficient at consuming fucosylated HMOs due to its lack of HMO-consumption related genes, including fucosidases, thus higher breast milk fucose content would be predicted to lead to lower colonization by *B. longum* subsp. *longum*^19^. *B. infantis* was the only microbe that was found to be positively correlated with LNnT, which confirms previous reports that *B. infantis* grows very well on this particular HMO species^18^. Thus the ability of the microbiome to degrade certain types of oligosaccharides is dependent on the specific strains comprising the infant’s microbiota. This strain variability may contribute to our failure to find functional differences in the microbiomes between infants fed by mothers of different secretor status using the predictive tool PICRUSt, as it is dependent on the specific sequenced strains available in public databases. Other factors must be taken into consideration when discussing the significance of these associations, such as cross-feeding between strains, growth of certain taxa on other microbial by-products, the influence of other breast milk components, and the fact that a known HMO-consuming bifidobacterial strain was dominant in the majority of these infants. Although our sequencing-based results do not test the HMO-consumption-related metabolic abilities and low-level enzymatic functions of the specific microbial species present in these infants, the correlations we present are in many cases consistent with the known abilities of studied strains. However, we cannot rule out the hypotheses that these observations are due to unrelated microbe-microbe interactions or co-correlation with a third factor rather than direct consumption of HMOs by the correlated microbe.

To evaluate the impact of microbial differences between classes of infants, the functional abilities of the fecal microbiome were predicted using PICRUSt. While no significant differences were found that might explain infant growth or health outcomes, three functions were found to increase over time. One of these functions, butyrate-acetoacetate CoA-transferase, can be involved in butyrate metabolism, and in the context of infant gastrointestinal tracts is likely involved in utilizing the larger concentrations of bifidobacterial-derived acetate present in the environment^45^,^60^,^61^,^62^,^63^. The largest contributor of this function was the Veillonellaceae. This shift likely reflects the maturation of the microbiome community as selective pressures assert themselves and niches are filled by better-adapted species.

Together these results lead us to conclude that changes in milk HMO composition, as well as shifts in the infant’s gut microbiota are both associated with infant health outcomes in The Gambia. Specifically, the relative abundance of LNFP I, the α(1-2) fucosylated structure of the parent compound LNT, was found to be higher in infants who did not have sick days compared to those who had sick days, and was also predictive of HAZ score at 20 weeks, suggesting that this HMO may be either directly or indirectly involved in protecting the infant from infection and thus promoting growth. The α(2-3) sialylated structure 3′SL was predictive for infant weight-for-age. Our findings suggest that specific HMOs and/or specific glycosyltransferase pathways can alter the composition of milk toward a more protective profile which is associated with lower rates of infection/inflammation, and which allow the infant to invest energy in growth. In this cohort, as with other cohorts in similar countries, infant gastrointestinal tracts were dominated by bifidobacteria. We were not able to detect statistically significant effects of shifts in bifidobacteria abundance because for most infants bifidobacteria levels stayed high (on average about 70% of total bacteria) across all three time points, although in five out of seven cases when bifidobacteria levels dropped significantly, there were concomitant increases in morbidity and/or growth faltering, as well as large shifts in HMO profiles. *Prevotella* was associated with decreased morbidity. In this study, we found that the seasonal fluctuations in energy availability in The Gambia affected the mother’s ability to produce HMOs such that mothers lactating during the wet (hungry) season had larger decreases in total HMOs over time than mothers lactating during the dry season. How the overall health, environment, and diet of the mother affects the quality of her milk and thus the ability of her milk to protect her infant from infection and promote growth is an important question to resolve in future studies. Mechanisms involved in mediating the protective effects of HMO profiles and specific HMO structures on infant morbidity and growth also need to be further studied, as do the gut microbiota-specific pathways involved in mediating these effects.

## Materials and Methods

### Experimental Design

The samples analyzed were from a sub-study embedded within a randomized trial to investigate the effects of pre-natal and infancy nutritional supplementation on infant immune development, The Early Nutrition and Immune Development (ENID) Trial, ISRCTN49285450, http://www.isrctn.com/ISRCTN49285450)^64^, registered on December 11, 2009. Ethical approval for the ENID Trial and the ‘ENID-Bioactives’ sub-study was obtained from the joint Gambian Government/MRC Unit The Gambia Ethics Committee and the George Washington University Institutional Research Board (#13-0441). Full informed consent was obtained from each participant, prior to inclusion in the study. Samples were collected from 200 Gambian mother-infant pairs at weeks 1, 4, 8, 12, 16, 20, 24, 28, 32, 36, 40, 44, 48, and 52 postpartum. Breast milk collection, infant anthropometry measurements, and infant morbidity data were collected according to a study design described previously^64^,^65^ and in accordance with relevant guidelines and regulations. Five mL of hand-expressed milk was collected from each breast into a separate tube in the morning during regular study visits at the Keneba clinic and the participants’ homes then stored at −80 °C. Mothers collected their infant’s fecal samples from a disposable diaper into a collection pot that was provided. The pot was then placed in a cold box with an ice pack and given to study personnel, who then delivered the sample to the Keneba clinic laboratory where samples were stored at −70 °C. Fecal samples were then freeze-dried for 24 hours and stored at −20 °C. For this study, a subset of 33 mothers and infants with samples at 4, 16, and 20 weeks was selected. After milk and fecal samples were shipped to UC Davis they were stored at −80 °C until analysis. Height, weight, and morbidity data of the infants were recorded weekly. Mothers self-reported morbidity data as observed incidents of illness for their infant. The days of illness for all of the disease categories were summed to create a new continuous variable of total number of sick days for each week. The number of sick days for each milk collection time point was calculated by summing the number of sick incidents for the four weeks surrounding that time point. Because milk composition changes over time, the four surrounding weeks were used to provide the best representation of how the HMO composition affected morbidity during that time frame. These values were used as a categorical value, designated as “sick” (if there were any sick days) or “not sick” (if there were no sick days during that time) (see Supplementary Table 2). Weight was measured using portable digital scales (Seca 334) with infants in minimal clothing. Length was measured using a portable length board (Seca 417). All measures were recorded in triplicate using standard protocols, and equipment was regularly calibrated. The weight and height of each infant at every time point were used to calculate weight-for-age (WAZ) and height-for-age (HAZ) Z scores based on a Gambian reference (see Supplementary Table 3). These scores were also grouped into categorical variables; infants with Z scores < −2 were deemed “underweight” or “stunted,” respectively, and those with scores > −2 were “desired weight” or “not stunted”^66^. See Supplementary Table 4 for further mother and infant clinical data.

### Infant Fecal DNA Extraction and Analysis

#### DNA extraction

DNA was extracted from the freeze-dried fecal samples using the ZR Fecal DNA MiniPrep Kit, (Zymo Research, Irvine, CA, USA) according to the manufacturer’s instructions. This included a bead-beating step using a FastPrep-24 Instrument (MP Biomedicals, Santa Ana, CA) for 2 minutes at 25 °C at a speed of 6.5 m/s. In a few cases, the default amount of lysis solution (750 μl) was insufficient to reconstitute the freeze-dried samples, and the addition of more (up to the capacity of the tube) was necessary to fully rehydrate the samples. Only 85 of the samples contained enough fecal matter to extract DNA for an analysis of the gut microbiota.

#### Sequencing and analysis

*Illumina sequencing—V4 region*: DNA extractions were prepared for marker gene sequencing as previously described^67^ with the following modifications. Universal barcoded primers with Illumina sequencing adapters (adapters are italicized and the barcode is highlighted in bold) V4F (5′-*AATGATACGGCGACCACCGAGATCTACACTCTTTCCCTACACGACGCTCTTCCGATCT* **ACTGCTGA**GTGTGCCAGCMGCCGCGGTAA-3′) and V4Rev (5′- *CAAGCAGAAGACGGCATACGAGATCGGTCTCGGCATTCCTGCTGAACCGCTCTTCCGATCT*CCGGACTACHVGGGTWTCTAAT-3′) were used to PCR amplify the V4 region of the 16S rRNA gene^67^. PCR reactions contained 7.5 μl 2x GoTaq Green Master Mix (Promega, Madison, WI), 0.6 μl 25 mM MgCl_2_, 3.6 μl water, 1.5 μl forward and 0.3 μl reverse primers (0.2 μM final concentration), and 1.5 μl DNA. A negative control was also included into which water was added in the place of DNA. A portion of each reaction was electrophoresed in a 0.8% agarose gel and stained with GelGreen (Phenix, Candler, NC). The DNA band for each sample was visually categorized by brightness and size for quality control. All samples were pooled (5 μl of each reaction for samples with bright bands, 10 μl for faint samples with bands, and 12 μl for samples with non-visible bands) and purified with the QIAquick PCR Purification Kit (QIAGEN, Valencia, CA) according to the manufacturer’s instructions. The pooled, purified amplicons were sequenced at the University of California-Davis DNA Technologies Core Facility on an Illumina MiSeq sequencing platform. A negative control was also included. The raw sequencing data and accompanying metadata were deposited in the European Nucleotide Archive under accession number ERP017462, and the Qiita database under Study ID 10297.

#### Sequence Analysis

The QIIME software package (version 1.8.0) was used to analyze the results of the Illumina sequencing run. llumina V4 16S rRNA gene sequences were demultiplexed and quality filtered using the QIIME 1.8 software package with default settings unless otherwise specified^67^. Reads were truncated after a maximum number of 3 consecutive low quality scores. The minimum number of consecutive high quality base calls to include a read (per single end read) as a fraction of the input read length was 0.75. The minimum acceptable Phred quality score was set at 20. Similar sequences were clustered into operational taxonomic units (OTUs) using open reference OTU picking with UCLUST software^68^. Taxonomy was assigned to each OTU with the Ribosomal Database Project (RDP) classifier^69^ and the RDP taxonomic nomenclature^70^. OTU representatives were aligned against the Greengenes core set^71^ with PyNAST software^72^.

#### Bif-TRFLP (terminal restriction fragment length polymorphism)

A *Bifidobacterium*-specific terminal restriction fragment length polymorphism assay was performed as previously described^73^. DNA from feces was PCR amplified using primers NBIF389 (5′-74-GCCTTCGGGTTGTAAAC) and NBIF1018 REV (GACCATGCACCACCTGTG). The Qiagen Qiaquick PCR purification kit DNA was used to clean the DNA, which was then cut with restriction enzymes AluI and HaeIII. The resulting fragments were analyzed on an ABI 3100 Capillary Electrophoresis Genetic Analyzer at the UC Davis College of Biological Sciences Sequencing Facility and compared against the published database for species identification.

#### Bifidobacterium Longum-Infantis Ratio (BLIR)

The BLIR method was used to quantify the relative amounts of two *B. longum* subspecies^25^. PCR was performed on the fecal DNA using three primers (FWD_BL_BI (5′-74-AAAACGTCCATCCATCACA), REV_BL (5′-ACGACCAGGTTCCACTTGAT), and REV_BI (5′-CGCCTCAGTTCTTTAATGT)) targeting a conserved portion of the genome (between Blon_0424 and Blon_0425) shared by both subspecies. FWD_BL_BI is complementary to a sequence in both subspecies while REV_BL and REV_BI are complementary to nearby sequences in only *B. longum* subsp. *longum* and *B. longum* subsp. *infantis*, respectively. FWD_BL_BI and REV_BL amplify a fragment of the *B. longum* spp. *longum* genome 145 bp in length, while FWD_BL_BI amplify a fragment of the *B. longum* subsp. *infantis* genome 114 bp in length.

DNA from each sample was amplified by PCR using 0.5 μl of 10 μM stock of each of the above primers, 12.5 μl GoTaq Green Master Mix (Promega), 1 μl of 25 mM MgCl_2,_ 1 μl of template DNA, and 9 μl of nuclease free water. Cycling conditions were 95 °C for 2 minutes, 30 cycles of 95 °C for 1 minute, 54 °C for 1 minute, and 72 °C for 30 seconds, followed by a 72 °C extension for 5 minutes. PCR products were purified from the mixture using the QIAquick PCR purification kit (Qiagen) and diluted 1:10. 1.5 μl of the dilutions were analyzed on an ABI 3100 Capillary Electrophoresis Genetic Analyzer at the UC Davis College of Biological Sciences Sequencing Facility. The samples were analyzed with PeakScanner 2.0 software (Applied Biosystems, Carlsbad, CA) to quantify peak area of amplicons from each subspecies. A positive control was included with each PCR run to ensure potential amplification of both *B. longum* subsp. *longum* and *B. longum* subsp. *infantis* products.

### Calprotectin Measurements

Intestinal inflammation was indexed by measuring infant fecal calprotectin levels. A commercial ELISA kit (Phical; CALPRO AS) was used, with the protocol adjusted for use on freeze-dried infant feces^75^. Calprotectin levels were also binned into three categories based on previously developed clinical diagnoses: normal (less than 50 mg/kg), borderline (50–120 mg/kg), and abnormal (greater than 120 mg/kg)^33^,^34^,^35^.

### HMO Extraction and Analysis

#### HMO extraction

Free HMOs were extracted from whole breast milk samples following previously reported methods^5^,^27^,^76^,^77^,^78^. 50 μL of each milk sample was aliquotted onto 96-well plates, diluted, and defatted via centrifugation. The resulting glycans were reduced with 1.0 M NaBH_4_ in a water bath at 65 °C for 1.5 hours. The samples were then purified on solid phase extraction graphitized carbon cartridges (GCC). HMO samples were loaded onto the GCCs, desalted with deionized water, and eluted with 20% acetonitrile in water and then 40% acetonitrile in 0.05% trifluoroacetic acid (v/v). The eluent fractions were combined and the solvent evaporated. After reconstitution, the samples were diluted to appropriate concentration for analysis.

#### Nano-HPLC-Chip/TOF Mass Spectrometry

Analysis of the extracted HMOs was performed on a nano-high performance liquid chromatography (HPLC)-chip/time-of-flight (TOF) mass spectrometer. The Agilent 1200 series HPLC (Agilent Technologies, Santa Clara, CA) unit incorporates a capillary pump for sample loading and a nano pump for analyte separation, done on a microfluidic chip. The chip contains a 40 nL enrichment column and a 75 μL × 43 mm analytical column packed with porous graphitized carbon. The HMO samples were loaded onto the enrichment column (1 μL injection) by the capillary pump at a flow rate of 4.0 μL/min. Separation was achieved with a binary gradient of aqueous solvent A (3% acetonitrile/water (v/v) in 0.1% formic acid) and organic solvent B (90% acetonitrile/water (v/v) in 0.1% formic acid). The method developed and optimized for separating HMO mixtures by Wu *et al*. was used^5^,^77^. This system is coupled to an Agilent 6220 series TOF mass spectrometer via chip-cube interface. Data was collected in the positive mode, and the instrument was calibrated by a dual nebulizer electrospray source with internal calibrant ions ranging from *m/z* 118.086 to 2721.895.

#### HMO Data Processing

Data was collected and analyzed using Agilent MassHunter Qualitative Analysis software, versions B.02.01 and B.03.01, respectively. HMO compounds were identified using the *Find Compounds by Molecular Feature* function, and an in-house program was used for peak alignment. Specific structures were identified and assigned by matching retention time and exact mass, within 20 ppm mass error, to theoretically calculated masses in previously developed annotated HMO libraries^5^,^77^. Absolute abundances in ion counts were directly correlated to the abundance of the compounds present. Glycan types were divided into four classes: fucosylated (any structure with Fuc), sialylated (any structure with Neu5Ac), fucosylated and sialylated, and undecorated. To determine the relative abundance of each class, abundances were divided by the total oligosaccharide count for each mother. The same calculation was performed to find relative abundances for individually identified compounds. Forty-three distinct structures were identified. If the peak abundance of an isomer could not be integrated separate from another closely eluting peak, those peak abundances were summed and the two structures grouped together.

#### Secretor Status Determination

The method used in this study for determining phenotypic secretor status was modified from a previously developed method for determining secretor status^27^. Absolute abundances of compounds with known α(1-2) linked Fuc (2′-fucosyllactose (2′FL), lactodifucotetraose (LDFT), trifucosyllacto-*N*-hexaose (TFLNH), difucosyllacto-*N*-hexaose a (DFLNHa), difucosyllacto-*N*-hexaose c (DFLNHc), and isomer I fucosyl-paralacto-*N*-hexaose (IFLNH I)^2^,^5^) were summed and normalized to each mother’s total oligosaccharide abundance to calculate relative α(1-2) fucosylation for mothers at all time points. Mothers with less than 6% relative α(1-2) fucosylation were assigned as phenotypic non-secretors; assigned secretor status was consistent at all time points.

### Statistical Analyses

#### HMO Analyses

All statistical analyses were done in R statistical software, unless specified otherwise (R core team, Vienna Austria). *P* ≤ 0.05 was considered significant. Absolute and relative oligosaccharide class and structure abundances were obtained for the 33 mothers at all 3 time points (*N* = 99). Analyses were performed using the abundances of HMO classes and individual structures found in at least 75% of all samples during at least two of the time points: LNT, LNFP I and III, LNnT, LSTc, MFLNH I and III, LSTb, MFpLNH IV, LNnH, LNH, DFLNHa, DFpLNH II, DFLNH b, 3′SL, 4021a and S-LNnH II, IFLNH III, and F-LNO. To determine if there were differences between secretors and non-secretors and infants who were “sick” and “not sick,” non-parametric Mann-Whitney U tests were performed on relative class abundances and relative abundances for the previously listed individual HMOs. Generalized Linear Mixed Effects modeling (GLME), using weeks as a repeated measure and the subjects as random effects, were used (*N* = 99) to determine differences across lactation weeks. Where appropriate, Tukey’s posthoc tests were used to identify individual differences. The Bartlett test was used to check for equal variance for all HMO class abundances; values were log_10_ transformed to meet homoscedasticity requirements for ANOVA tests. Pearson’s correlation analyses were used to determine associations between top twelve most abundant microbiota, relative *B. longum* subsp. *longum* and *B. longum* subsp. *infantis* with absolute HMO abundances (*N* = 85) using JMP Pro 12.1 (SAS Institute, Cary, NC). Area under the curve (AUC) for all data were calculated using the trapezoidal method. The AUC for relative class abundances and relative individual HMO structures listed above were entered into a stepwise linear regression model (backwards using AIC criterion) to determine which among these variables best predict variation in HAZ and WAZ scores at week 20.

#### Microbiota Analyses

Only 85 of the total 99 samples contained enough fecal matter to extract DNA for an analysis of the gut microbiota. The marker gene sequencing data was tested using LefSe (LDA Effect Size) with default settings (unless otherwise noted) to investigate differences between classes of samples^79^. For PICRUSt, the OTU table from QIIME was filtered to contain just the closed-reference-picked OTUs and normalized by predicted 16S copy numbers. The KEGG Ortholog (KO) metagenome was then predicted by PICRUSt using the predict_metagenomes.py script^80^. The resulting KO table was then investigated for differences in the predicted functional profile of the fecal microbiome between classes of samples using the Statistical Analysis of Metagenome Profiles (STAMP) implementation of the Kruskal-Wallis H-test with a Benjamini-Hochberg False Discovery Rate (FDR) correction^81^,^82^.

## Additional Information

**Accession codes:** The microbiome sequencing data have been uploaded to the online Qiita database (www.qiita.microbio.me) under Study ID 10297 and placed in the European Nucleotide Archive under accession number ERP017462.

**How to cite this article:** Davis, J. C. C. *et al*. Growth and Morbidity of Gambian Infants are Influenced by Maternal Milk Oligosaccharides and Infant Gut Microbiota. *Sci. Rep.* **7**, 40466; doi: 10.1038/srep40466 (2017).

**Publisher's note:** Springer Nature remains neutral with regard to jurisdictional claims in published maps and institutional affiliations.

## Supplementary Material

Supplementary Materials

## Figures and Tables

**Table 1:** Identified HMO structures and their relative abundances at each time point.

Structure	Relative Abundance (%)			Number of Samples Found In		
	Week 4	Week 16	Week 20	Week 4	Week 16	Week 20
LNTa,^b^	23.8	21.3	22.7	33	33	33
LNFP I + III	10.3	10.5	10.2	25	29	28
LNnT	8.68	10.9	10.7	18	26	25
LSTca,b,c,^d^	4.86	3.26	3.76	33	33	33
MFLNH I + III^b^	3.82	2.87	3.23	24	29	32
MFpLNH IV	2.57	3.71	2.93	32	30	31
LSTb	2.29	3.19	3.16	33	33	33
LNnH	2.44	2.46	2.32	33	33	33
DFLNHa	2.48	2.14	2.50	30	30	28
LNHa,b,c,^d^	3.50	1.72	1.67	33	31	33
DFpLNH II	1.68	2.81	2.20	27	27	28
DFLNH bc,^d^	1.24	2.19	2.71	28	25	25
3′SLb,c,^d^	0.776	1.25	1.38	33	33	33
4021a + S-LNnH II	1.19	1.07	0.986	32	28	29
IFLNH III	1.03	1.03	1.01	31	29	29
F-LNO	0.712	0.522	0.527	29	26	26
2′FL	7.66	11.8	9.85	22	23	25
LNDFH II	3.55	3.34	3.97	19	23	21
LDFT	1.97	4.27	3.88	24	24	25
4120a	2.16	3.46	2.51	16	11	14
TFLNH	1.52	1.80	1.72	18	18	18
p-LNH	1.69	1.57	1.36	23	21	23
F-LSTc	1.16	1.48	1.16	23	18	19
LNDFH I	0.547	1.34	0.907	5	9	10
5130a	1.36	0.669	0.726	27	22	22
5230a + DFLNnO I/DFLNO II	0.968	0.592	0.652	26	24	24
5330a	0.741	0.700	0.647	17	10	11
IFLNH I	0.709	0.621	0.721	22	20	20
DFLNnO II	1.01	0.468	0.550	28	23	23
DFLNO I	0.842	0.571	0.548	27	18	20
5130b	0.673	0.518	0.420	16	8	8
S-LNH	0.815	0.305	0.356	33	15	15
LSTa	0.341	0.355	0.431	24	25	25
LNFP II	0.355	0.362	0.318	18	22	21
6′SL	0.370	0.288	0.243	26	16	15
DFLNHc	0.237	0.204	0.322	13	5	6
5130c	0.277	0.283	0.200	17	6	9
4320a	0.224	0.247	0.217	8	5	6
3FL	0.164	0.263	0.249	9	15	14

^a^Significant differences in absolute abundances between week 4 and week 16.

^b^Significant differences in absolute abundances between week 16 and week 20.

^c^Significant differences in relative abundances between week 4 and week 16.

^d^Significant differences in relative abundances between week 16 and week 20.

**Table 2:** Relative abundances of the most prevalent microbiota over the infants’ first 20 weeks of life.

OTU								Average Abundance (%)
k__Bacteria;p__Actinobacteria;c__Actinobacteria;o__Bifidobacteriales;f__Bifidobacteriaceae;g__Bifidobacterium								68.3
k__Bacteria;p__Firmicutes;c__Bacilli;o__Lactobacillales;f__Streptococcaceae;g__Streptococcus								7.72
k__Bacteria;p__Proteobacteria;c__Gammaproteobacteria;o__Enterobacteriales;f__Enterobacteriaceae;g__								5.41
k__Bacteria;p__Firmicutes;c__Clostridia;o__Clostridiales;f__Veillonellaceae;g__Megasphaera								2.37
k__Bacteria;p__Bacteroidetes;c__Bacteroidia;o__Bacteroidales;f__Bacteroidaceae;g__Bacteroides								2.02
k__Bacteria;p__Proteobacteria;c__Epsilonproteobacteria;o__Campylobacterales;f__Campylobacteraceae;g__Campylobacter								1.80
k__Bacteria;p__Firmicutes;c__Bacilli;o__Lactobacillales;f__Lactobacillaceae;g__Lactobacillus								1.78
k__Bacteria;p__Actinobacteria;c__Coriobacteriia;o__Coriobacteriales;f__Coriobacteriaceae;g__								1.55
k__Bacteria;p__Bacteroidetes;c__Bacteroidia;o__Bacteroidales;f__Porphyromonadaceae;g__Parabacteroides								1.51
k__Bacteria;p__Bacteroidetes;c__Bacteroidia;o__Bacteroidales;f__Prevotellaceae;g__Prevotella								1.25
k__Bacteria;p__Firmicutes;c__Clostridia;o__Clostridiales;f__Veillonellaceae;g__Veillonella								1.02
k__Bacteria;p__Proteobacteria;c__Betaproteobacteria;o__Burkholderiales;f__Alcaligenaceae;g__Sutterella								0.461
k__Bacteria;p__Firmicutes;c__Clostridia;o__Clostridiales;f__[Mogibacteriaceae];g__								0.407
k__Bacteria;p__Firmicutes;c__Bacilli;o__Lactobacillales;f__Enterococcaceae;g__Enterococcus								0.315
k__Bacteria;p__Firmicutes;c__Bacilli;o__Bacillales;f__Staphylococcaceae;g__Staphylococcus								0.187
k__Bacteria;p__Firmicutes;c__Erysipelotrichi;o__Erysipelotrichales;f__Erysipelotrichaceae;g__								0.170
k__Bacteria;p__Firmicutes;c__Clostridia;o__Clostridiales;f__;g__								0.148
k__Bacteria;p__Verrucomicrobia;c__Verrucomicrobiae;o__Verrucomicrobiales;f__Verrucomicrobiaceae;g__Akkermansia								0.132
k__Bacteria;p__Proteobacteria;c__Betaproteobacteria;o__Neisseriales;f__Neisseriaceae;g__Neisseria								0.131
k__Bacteria;p__Firmicutes;c__Bacilli;o__Lactobacillales;f__Aerococcaceae;g__Facklamia								0.111
k__Bacteria;p__Tenericutes;c__Mollicutes;o__Mycoplasmatales;f__Mycoplasmataceae;g__								0.0771
All Others								3.15
Bifidobacerial species	*B. adolescentis*	*B. animalis*	*B. breve*	Unidentified *B. longum* group	*B. longum* subsp. *infantis*	*B. longum* subsp. *longum*	*B. bifidum/B. pseudocatenulatum*	Unknown
Percent of total bifidobacteria	0	0.0127	0.941	2.22	76.9	14.1	5.69	0.179

**Table 3:** Multiple Linear Regression stepwise models used to predict weight-for-age (WAZ) and height-for-age (HAZ) Z scores at 20 weeks postpartum.

Parameter	Final model predictors (Log transformed)	Coefficient	F statistic	Multiple R^2^	Overall *P*-value
WAZ	%3′SL	1.7886	7.808	0.3374	0.8 × 10^−6^
	%LSTc	−1.1185			
HAZ	%LNFP I + III	0.5670	2.459	0.0947	0.05
	%DFLNHa	0.4995			
