# Supplementary material for: Growth and Morbidity of Gambian Infants are Influenced by Maternal Milk Oligosaccharides and Infant Gut Microbiota
Source: Sci Rep. 2017 Jan 12;7:40466. doi: 10.1038/srep40466 (PMC5227965; doi:10.1038/srep40466)

Growth and Morbidity of Gambian Infants are Influenced by Maternal Milk Oligosaccharides and Infant Gut Microbiota

Authors: Jasmine C. C. Davis[1,2], Zachery T. Lewis[2,3], Sridevi Krishnan[4], Robin M. Bernstein[6,7], Sophie E. Moore[8,9], Andrew M. Prentice[9], David A. Mills[2,3,5], Carlito B. Lebrilla[1,2], and Angela M. Zivkovic[2,4]*

Affiliations:

[1]Department of Chemistry, [2]Foods for Health Institute, [3]Department of Food Science and Technology, [4]Department of Nutrition, and [5]Department of Viticulture and Enology, University of California, Davis, CA 95616, United States

[6]Department of Anthropology, [7]Health and Society Program, Institute of Behavioral Science, University of Colorado, Boulder, CO 80309, United States

[8]Medical Research Council (MRC) Human Nutrition Research, Cambridge, UK

[9]MRC Unit, The Gambia and MRC International Nutrition Group, London School of Hygiene & Tropical Medicine, London, UK

*To whom correspondence should be addressed: Angela M. Zivkovic, amzivkovic@ucdavis.edu

Supplementary Figure Legends

Supplementary Figure S1. Differences in individual human milk oligosaccharides (HMOs) between infants who were sick and those who were not sick. Plots are divided by sick (■) vs. not sick (■) infants, displaying average and standard error bars for relative abundances of (**A**) F-LNO (fucosyllacto-*N*-octaose) at week 4, (**B**) MFpLNH IV (fucosyl-para-lacto-*N*-hexaose) at week 16, and (**C**) IFLNH III (isomer III fucosyl-paralacto-*N*-hexaose) at week 16. *P*-value based on Mann-Whitney U tests. HMO structures pictured with monosaccharides Glc (●), Gal (●), GlcNAc (■), and Fuc (▲).

Supplementary Figure S2. Relative abundance plots showing the differences in the gut microbiota between infants who were sick and those who were not. The plots show the class-wise distribution of the enriched taxa at the lowest taxonomic level at which they are enriched.

Supplementary Figure S3. Box plots displaying change in infant fecal calprotectin levels (mg/kg) at 4, 16, and 20 weeks postpartum. Median is given as a line, 25th and 75th percentiles are the top and bottom of the box, whiskers are data within 1.5*(75th – 25th percentile) of the median, outliers displayed as dots above the box. *N* at each week given above plot with *P*-value based on linear mixed effects model.

Supplementary Figure S4. Relative abundance plots showing the differences in the gut microbiota between infants categorized into having normal, borderline, or abnormal calprotectin levels. The plots show the class-wise distribution of the enriched taxa at the lowest taxonomic level at which they are enriched.

Supplementary Figure S5. Cladogram plots of microbiota enriched in infants categorized by (**A**) WAZ and (**B**) HAZ scores. Infants with WAZ scores <-2 were assigned as "underweight,"

infants with scores >-2 were "desired weight," infants with HAZ scores <-2 were assigned as "stunted," and infants with scores >-2 were "not stunted." Plots indicate microbes that were enriched in infants who were at desired weight (■) or underweight (■), and not stunted (■) or stunted (■) based on LefSe analysis.

Supplementary Figure S6. Cladogram plot of microbiota enriched in infants born in either the dry (■) or wet (■) season.

Supplementary Figure S7. Changes in the predicted functional profile of the infant fecal microbiome over time from week 4 (■) to week 16 (■) to week 20 (■). The median of the data is shown as a line, the mean of the data as a star, the 25th and 75th percentiles of the data as the top and bottom of the box, and the most extreme data point within 1.5*(75th – 25th percentile) of the median as whiskers. Data points outside of the whiskers are shown as crosses.

Supplementary Figure S8. Associations between growth, morbidity, and a decrease in bifidobacteria abundance for infant 25. From week 16 to week 20, the relative abundance of bifidobacteria (■) severely dropped and infant calprotectin (■) severely increased. Infant 25 also had 12 morbidity instances (■), unlike weeks 4 and 16 in which she experienced zero. Her WAZ score (■) also dropped during these time points.

Supplementary Table 1. Major microbial contributors of metabolic functions found to change over the first 20 weeks of life.

Gene	Order	Family	Genus	Proportional contribution in all samples
Hexokinase	Bacteroidales	Bacteroidaceae	Bacteroides	0.286
Hexokinase	Clostridiales	Veillonellaceae	Megasphaera	0.418
Hexokinase	Bacteroidales	Porphyromonadaceae	Parabacteroides	0.293
Butyrate-acetoacetate CoA-transferase	Clostridiales	Veillonellaceae	Megasphaera	0.953
Butyrate-acetoacetate CoA-transferase	Fusobacteriales	Fusobacteriaceae	Fusobacterium	0.0339
Butyrate-acetoacetate CoA-transferase	Clostridiales	Lachnospiraceae	All	0.0136
Glutaconyl-CoA decarboxylase	Clostridiales	Veillonellaceae	All	0.932
Glutaconyl-CoA decarboxylase	Clostridiales	Lachnospiraceae	All	0.0336
Glutaconyl-CoA decarboxylase	Clostridiales	Clostridiaceae	All	0.0161

Supplementary Table 2. Morbidity instances used to assign infants to "sick" or "not sick" categories.

Sample	Number of Morbidity Instances			Sick or Not Sick		
	Week 4	Week 16	Week 20	Week 4	Week 16	Week 20
1	0	13	0	Not Sick	Sick	Not Sick
2	7	1	0	Sick	Sick	Not Sick
3	15	2	0	Sick	Sick	Not Sick
4	5	0	0	Sick	Not Sick	Not Sick
5	0	0	11	Not Sick	Not Sick	Sick
6	28	0	0	Sick	Not Sick	Not Sick
7	9	7	24	Sick	Sick	Sick
8	0	1	0	Not Sick	Sick	Not Sick
9	0	13	9	Not Sick	Sick	Sick
10	5	0	0	Sick	Not Sick	Not Sick
11	0	0	0	Not Sick	Not Sick	Not Sick
12	0	3	5	Not Sick	Sick	Sick
13	0	9	0	Not Sick	Sick	Not Sick
14	0	0	0	Not Sick	Not Sick	Not Sick
15	8	0	5	Sick	Not Sick	Sick
16	4	0	5	Sick	Not Sick	Sick
17	8	5	8	Sick	Sick	Sick
18	4	4	15	Sick	Sick	Sick
19	0	0	0	Not Sick	Not Sick	Not Sick
20	10	0	7	Sick	Not Sick	Sick
21	0	0	0	Not Sick	Not Sick	Not Sick
22	0	9	19	Not Sick	Sick	Sick
23	0	18	11	Not Sick	Sick	Sick
24	0	9	0	Not Sick	Sick	Not Sick
25	0	0	12	Not Sick	Not Sick	Sick
26	0	0	3	Not Sick	Not Sick	Sick
27	4	0	0	Sick	Not Sick	Not Sick
28	0	0	9	Not Sick	Not Sick	Sick
29	0	7	0	Not Sick	Sick	Not Sick
30	0	5	6	Not Sick	Sick	Sick
31	0	4	0	Not Sick	Sick	Not Sick
32	0	0	0	Not Sick	Not Sick	Not Sick
33	0	0	0	Not Sick	Not Sick	Not Sick

Supplementary Table 3. Weight-for-age (WAZ) and height-for-age (HAZ) Z scores calculated for each infant against a Gambian reference.

Sample	WAZ score			HAZ score		
	Week 4	Week 16	Week 20	Week 4	Week 16	Week 20
1	0.167	-1.18	-1.18	0.909	-0.322	-1.77
2	-1.85	0.338	0.913	0.728	-3.76	0.736
3	-0.341	-0.0796	-0.146	0.292	0.157	-0.575
4	-1.67	0.926	0.863	-0.613	0.367	0.639
5	0.587	2.78	2.75	1.12	-1.54	1.58
6	-1.61	1.28	0.875	0.536	2.04	0.736
7	0.0952	0.625	0.202	-0.133	0.591	0.487
8	0.389	0.318	0.281	1.12	0.243	1.45
9	-1.72	-0.0539	-0.1	-0.153	-0.898	-1.19
10	-1.9	0.0294	0.1	0.69	0.735	0.351
11	-1.75	0.25	0.129	0.728	0.816	0.527
12	0.143	0.284	0.101	0.21	0.157	0.398
13	1.32	1.55	1.31	1.73	1.24	1.46
14	-2.38	-0.662	-0.65	0.345	0.531	1.65
15	0.452	1.09	1.29	0.58	0.388	0.929
16	0.706	1.51	2.09	0.704	1.46	1.37
17	0.296	-1.57	-0.35	1.53	0.816	0.0625
18	-2.58	0.706	-1	-0.996	-1.51	-0.995
19	-0.579	-1.02	-0.708	-0.449	-0.974	-0.974
20	-2.39	0.19118	0.0625	0.345	0.816	1.41
21	-2.15	-0.926	-0.617	0.345	-1.1	-0.354
22	-1.54	1.43	1.5	1.8	1.14	0.351
23	0.23	0	0.397	0.498	0.809	0.221
24	0.238	-0.739	-0.124	0.704	0.722	0.487
25	-1.48	-0.0882	-0.35	0.536	0.939	1.31
26	-0.286	-0.432	-0.303	0.498	0.2	-0.398
27	-2.77	-1.41	-1.05	-2.15	-1.92	-2.82
28	-2.66	-0.99	-0.85		-0.286	-0.995
29	-1.79	-1.63	-1.41		-0.408	-0.611
30	-0.317	0.837	0.652	-0.901	-0.409	-0.664
31	0.341	0.409	0.27	-0.531	0.678	-0.693
32	0.516	0.443	0.506	1.16	1.2	0.531
33	-0.381	-1.25	-0.727	-1.77	-2.67	-2.45

Supplementary Table 4. Maternal age and weight, and infant sex, weight, and height.

Sample	Maternal Age (years)	Maternal Weight (kg)	Infant Sex	Infant Weight (kg)			Infant Height (cm)		
		Week 1		Week 4	Week 16	Week 20	Week 4	Week 16	Week 20
1	42	54.37	M	4.61	5.66	5.8	56.5	62.9	60.9
2	22	73.6	F	4.25	6.36	7.03	55.5	53	64.8
3	21	51.27	M	3.97	6.63	6.72	55	64	63.6
4	23	47.6	F	4.43	6.76	6.99	52	63.1	64.6
5	34	57.07	M	5.14	9.15	9.29	57	60.1	68.47
6	33	60.9	F	4.49	7	7	55	67.2	64.8
7	27	55.3	M	4.52	7.25	7.03	53.97	65	66
8	37	53.7	M	4.89	6.98	7.1	57	64.2	68.2
9	30	54.83	F	4.38	6.09	6.22	53.2	60	60.8
10	32	66.33	F	4.2	6.15	6.38	55.4	64	64
11	33	58.83	F	4.35	6.3	6.4	55.5	64.2	64.37
12	23	57	M	4.58	6.95	6.94	54.8	64	65.8
13	44	61.8	M	6.06	8.06	8.02	58.5	66.5	68.2
14	40	58.77	F	3.72	5.68	5.78	54.5	63.5	66.7
15	24	60.4	M	4.97	7.66	8	55.7	64.53	67
16	21	50.83	M	5.29	8.03	8.71	56	67	68
17	28	58.33	F	6.4	5.06	6.02	57.6	64.2	63.4
18	22	56.37	F	3.52	6.61	5.5	51	58.5	61.2
19	29	60.7	M	3.67	5.8	6.22	53.2	61.4	62.7
20	28	86.73	F	3.71	6.26	6.35	54.5	64.2	66.2
21	43	57.4	F	3.95	5.5	5.81	54.5	59.5	62.53
22	31	55.8	F	4.56	7.1	7.5	58.3	65	64
23	25	53.9	M	4.69	6.7	7.2	55.5	65.5	65.4
24	43	61.9	M	4.7	6.05	6.74	56	65.3	66
25	37	59.57	F	4.62	6.07	6.02	55	64.5	66
26	28	56.77	M	4.04	6.32	6.58	55.5	64.1	64
27	30	48.9	F	3.33	5.17	5.46	48	57.5	57.4
28	24	48.77	F	3.44		5.62	51	61.5	61.2
29	34	59.8	F	4.31		5.17	59	61.2	62
30	19	62.27	M	4	7.44	7.43	52.1	62.7	63.4
31	24	53.6	M	4.83	7.06	7.09	53	65.2	63.33
32	21	54.3	M	5.05	7.09	7.3	57.1	66.4	66.1
33	45	60.63	M	3.92	5.6	6.2	50	57.5	59.37

Supplementary Figure S1. Differences in individual human milk oligosaccharide (HMO) structures between "sick" and "not sick" infants.

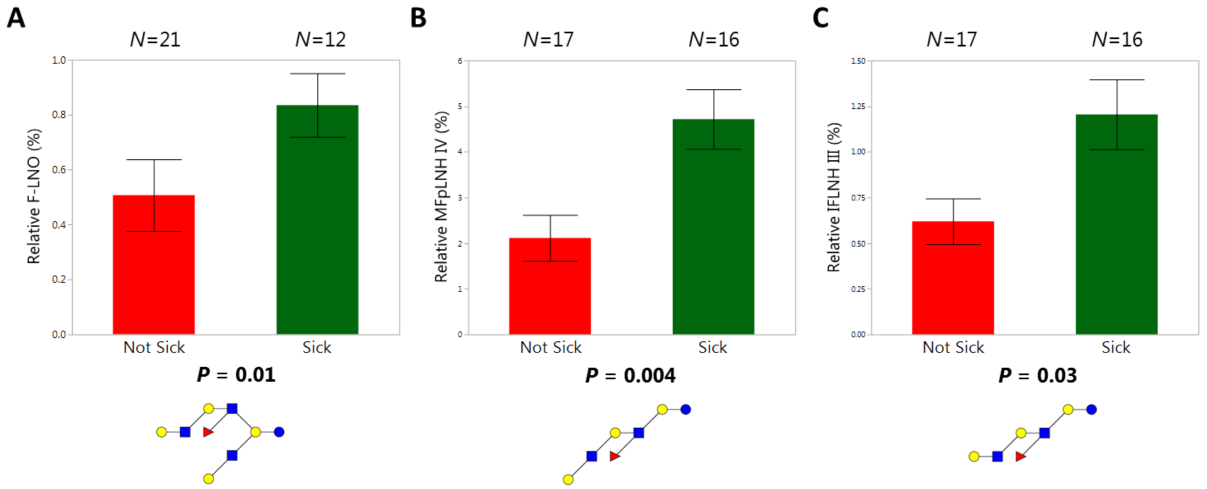

Supplementary Figure S2. Relative abundance plots of enriched taxa of infants who were classified as “sick” or “not sick.”

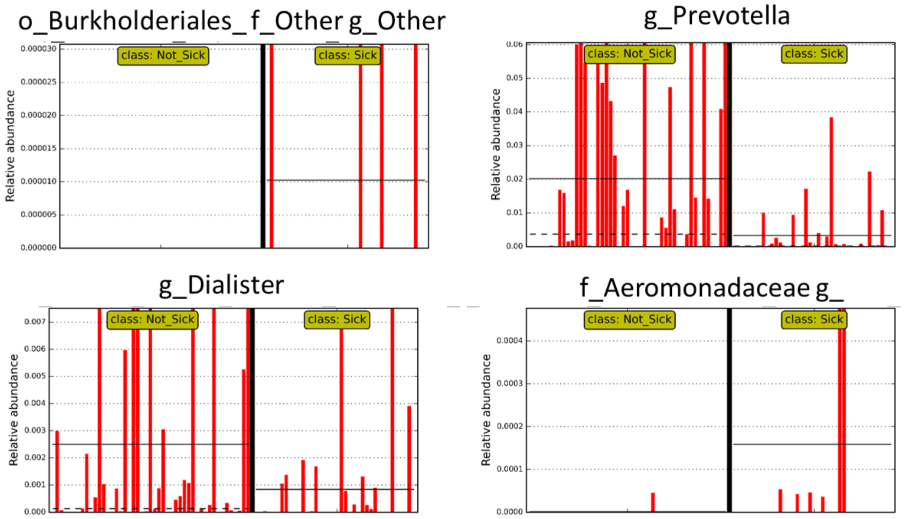

Supplementary Figure S3. Infant fecal calprotectin levels by week.

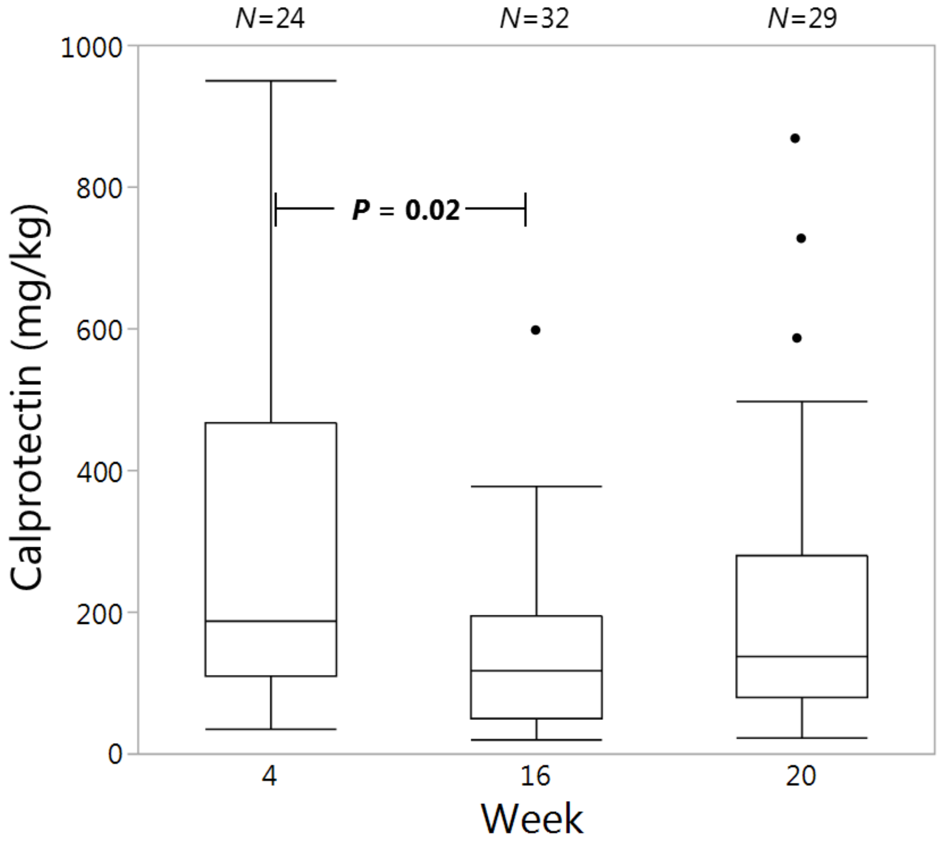

Supplementary Figure S4. Relative abundance plots of enriched taxa of infants with normal, borderline, and abnormal calprotectin levels.

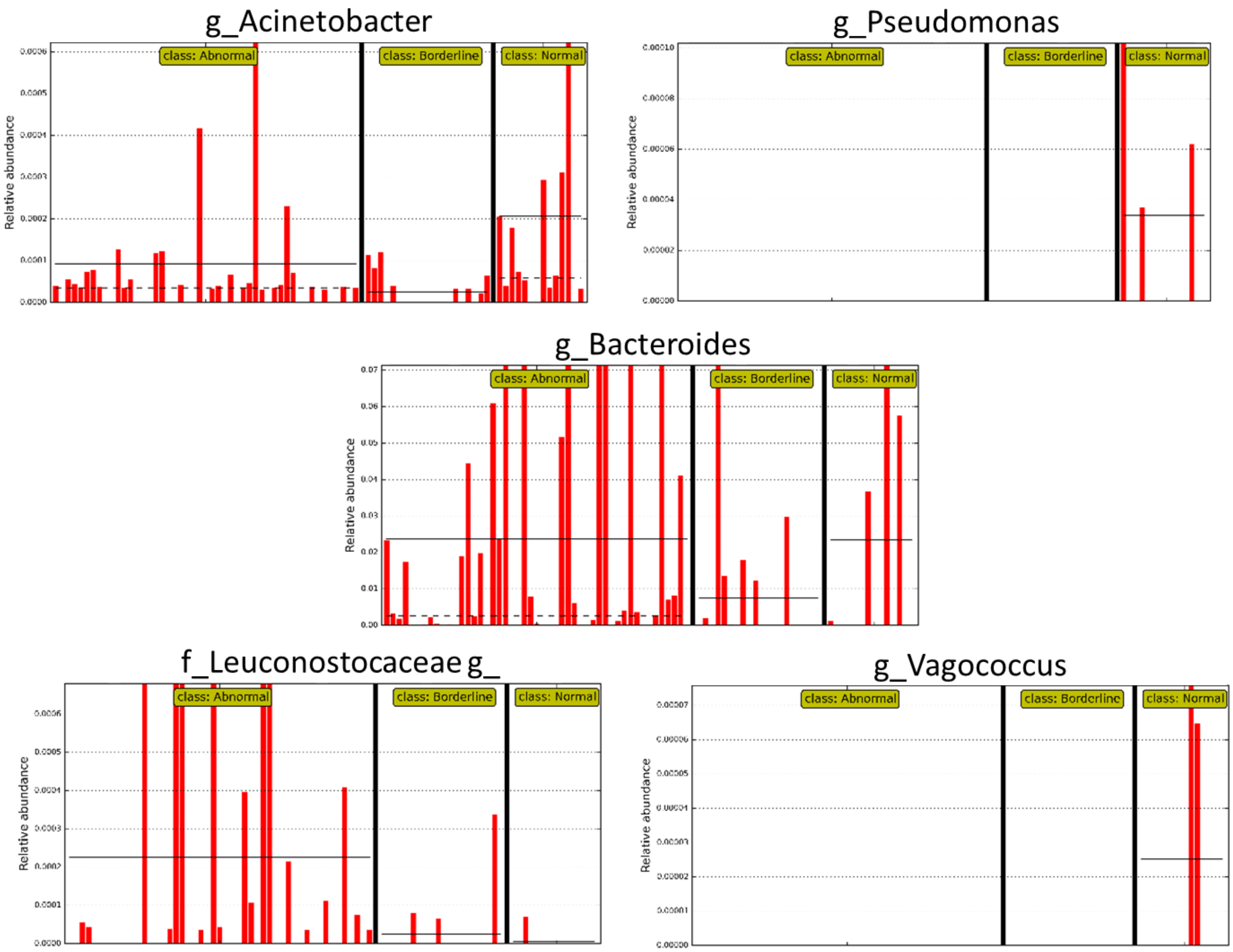

Supplementary Figure S5. Cladograms of microbiota enriched in infants separated by growth outcomes.

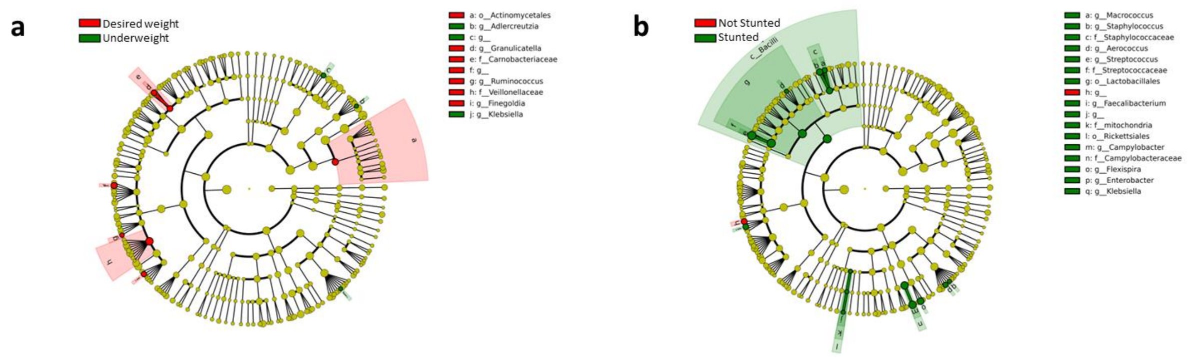

Supplementary Figure S6. Cladograms of microbiota enriched in infants based on birth season.

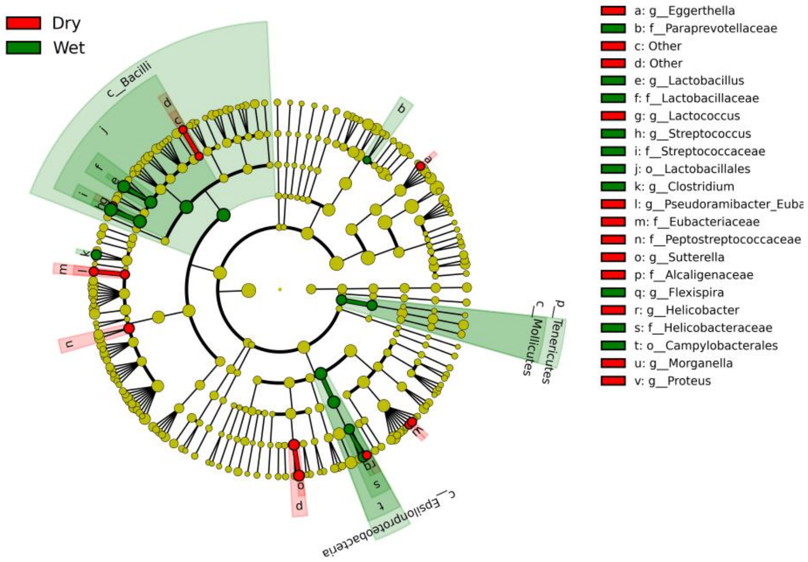

Supplementary Figure S7. Changes in predicted functional profiles of microbiota over time.

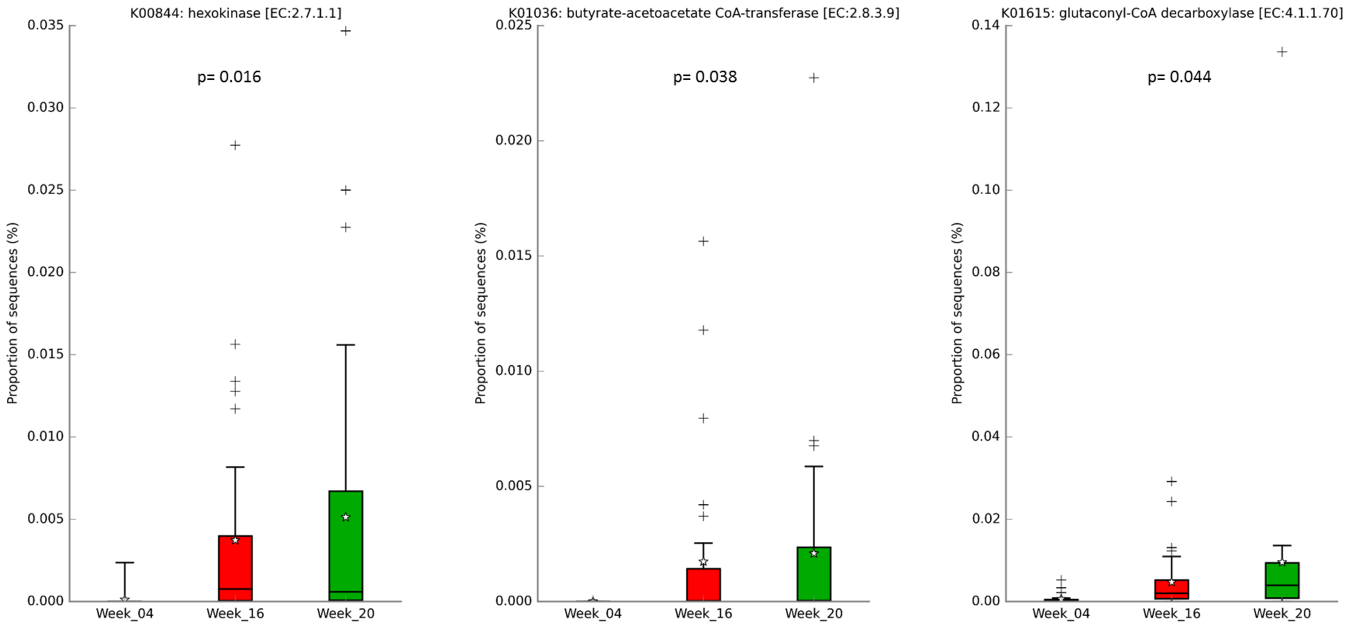

Supplementary Figure S8. Associations between growth faltering and increased morbidity with a decrease in abundance of bifidobacteria.

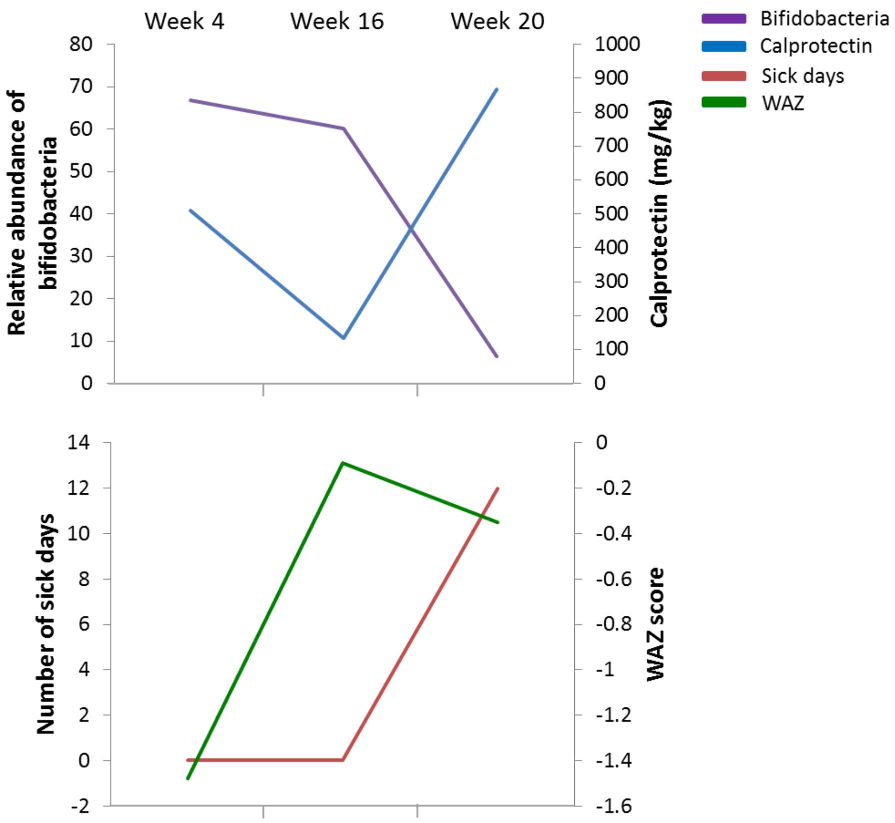

Supplement: Supplementary Materials [file srep40466-s1.pdf]
